# Supplementary material for: Cellular miR-6741-5p as a Prognostic Biomarker Predicting Length of Hospital Stay among COVID-19 Patients
Source: Viruses. 2022 Nov 30;14(12):2681. doi: 10.3390/v14122681 (PMC9781286; doi:10.3390/v14122681)
Supplement: Supplementary file 1 [file viruses-14-02681-s001.zip › viruses-1969111-supplementary.pdf]

SUPPLEMENTAL SECTION

Cellular miR-6741-5p as a Prognostic Biomarker Predicting Length of Hospital Stay among COVID-19 Patients

Shaw M. Akula [1,2,*], John F. Williams [1], Lok R. Pokhrel [3], Anais N. Bauer [1], Smit Rajput [2] and Paul P. Cook [2]

[1] Department of Microbiology & Immunology, Brody School of Medicine, East Carolina University, Greenville, NC 27834, USA

[2] Department of Internal Medicine, Brody School of Medicine, East Carolina University, Greenville, NC 27834, USA

[3] Department of Public Health, Brody School of Medicine, East Carolina University, Greenville, NC 27834, USA

* Correspondence: akulas@ecu.edu

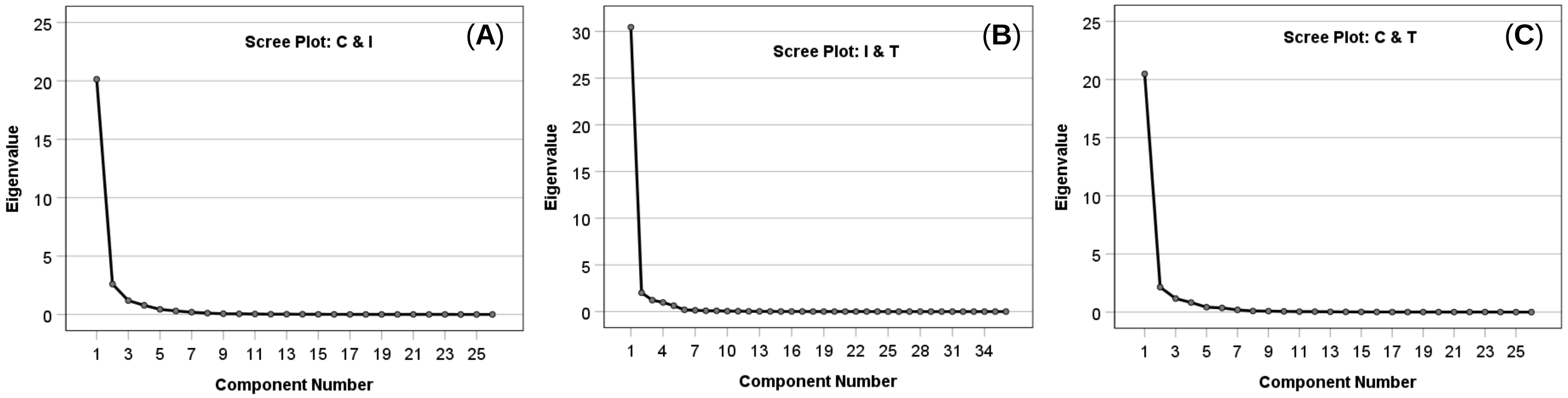

Supplementary Figure S1. Scree plots showing eigenvalues for the principal component presented in Figure 1. (**A-C**) Scree Plot showing eigenvalues on the Y-axis and the number of factors on the X- axis for transcriptome differences between miRNA expression in I, T, and C groups of COVID-19 patients. All three scree plots show that the first two factors (principal components [PC] 1 and 2) are to be considered in the principal component analysis, and accordingly we have plotted PC1 vs PC2 between the groups: C & I (**A**), I & T (**B**), and C & T (**C**) in Figure 1 in the main text. **C** denotes healthy volunteers, **I** denotes COVID-19 patients treated with remdesivir (an antiviral) plus dexamethasone (a glucocorticoid) (with or without baricitinib, a Janus kinase inhibitor) on the day of hospitalization, and **T** denotes COVID-19 patients at 48h post treatment.

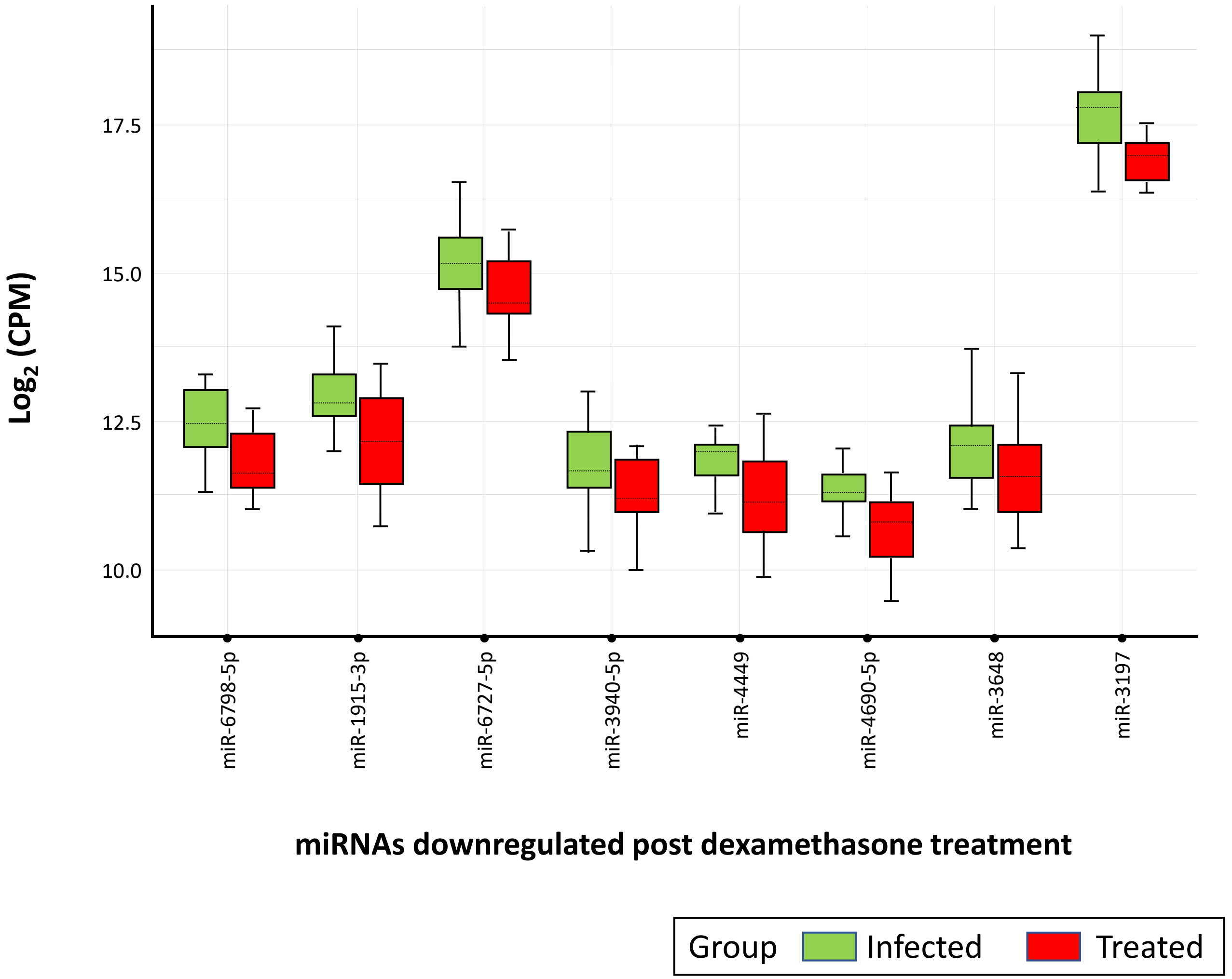

Supplementary Figure S2. Boxplots of the 8 miRNAs downregulated in COVID-19 patients with moderate-severe illness at 48h post treatment. Screening of 2083 human miRNA transcripts using extraction-free HTG EdgeSeq system determined 8 differentially expressed circulating miRNAs in the plasma of COVID-19 patients on the day of hospitalization (Infected) and 48h post treatment (Treated).

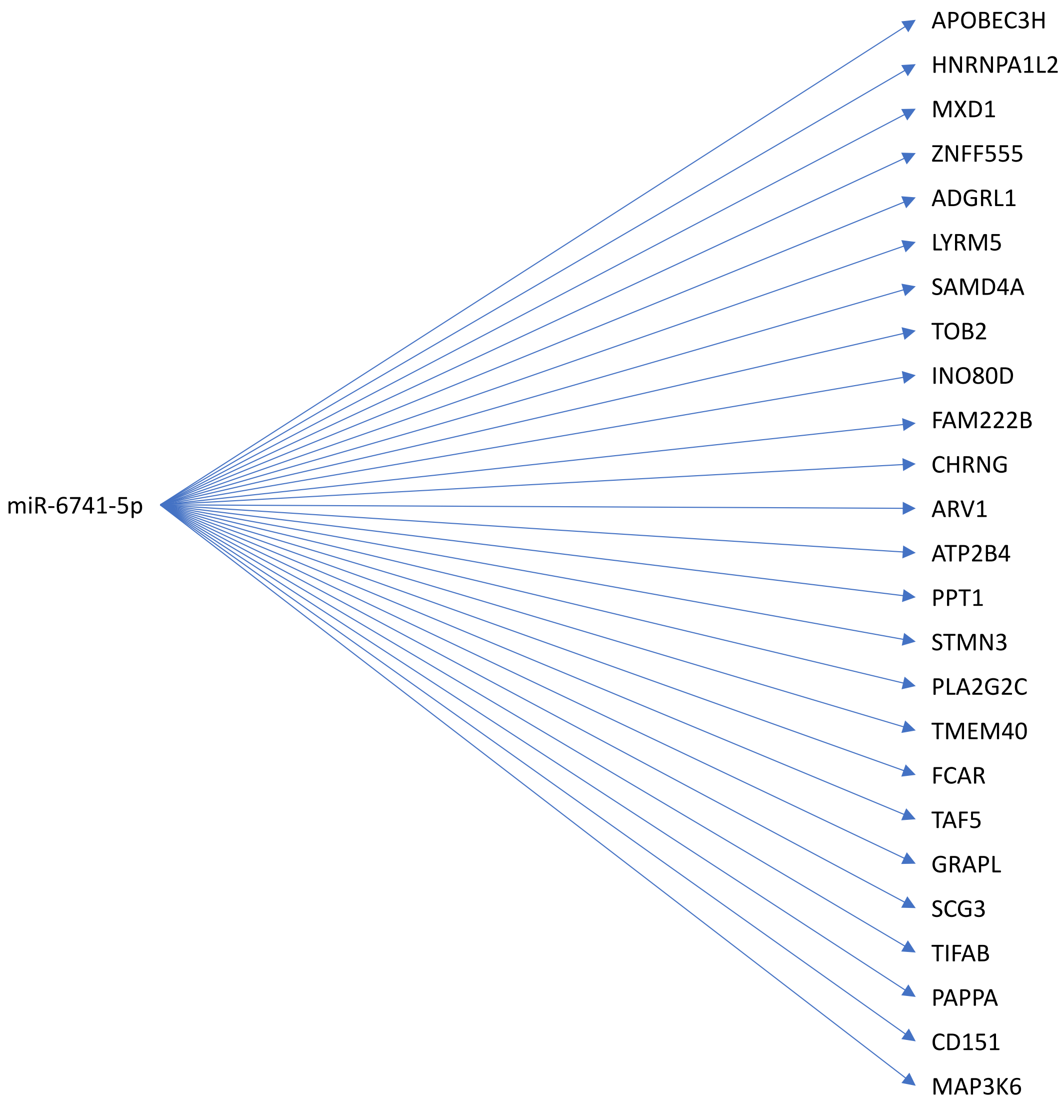

Supplemental Figure S3. Putative target genes for miR-6741-5p. miR-6741-5p has been predicted to modulate expression of at least 25 target genes. miRDB and TargetScan tools were used for prediction of miR-6741-5p target genes.

Position 269-275 of HNRNPA1L2 3' UTR

hsa-miR-6741-5p

```
5' ...UAGAUUUUUUUUUUUGCACCCAU...
                     |||||||
3'     GUGCCGAGGGUGGUCGUGGGUG
```

Position 24-31 of APOBEC3H 3' UTR

hsa-miR-6741-5p

```
5' ...AUCUACUGGAACAUAGCACCCAA..
                     |||||||
3'     GUGCCGAGGGUGGUCGUGGGUG
```

Supplementary Figure S4. RNA hybrid analysis shows miR-6741-5p binding site in 3;-UTR of HNRNPA1L2 and APOBEC3H mRNA. This is predicted using miRDB and TargetScan algorithms [14,15].

Supplementary Table S1: Oligos used in the luciferase assay.

Name	Sequence
hsa-miR-6741-5p mimic	gugggugcuggugggagccgug
miR-NC	ucacaaccuccuagaaagaguaga
WT human HNRNPA1L2 3'UTR sequence	AAAGTGttttaatgtagattttttttttgcacccat..........
WT human APOBEC3H 3'UTR sequence	CTCGAGgtcacccaatctactggaacatagcacccaa..........
human APOBEC3H 3'UTR Mutant-1	CTCGAGGtacccaactctactggaacatagcacccaa..........
human APOBEC3H 3'UTR Mutant-2	CTCGAGGtcacccaatctactggaacatacaaacgcc..........
human APOBEC3H 3'UTR Mutant-1&2	CTCGAGGtacccaactctactggaacatacaaacgcc..........

miRNA binding sequence

Mutated sequence

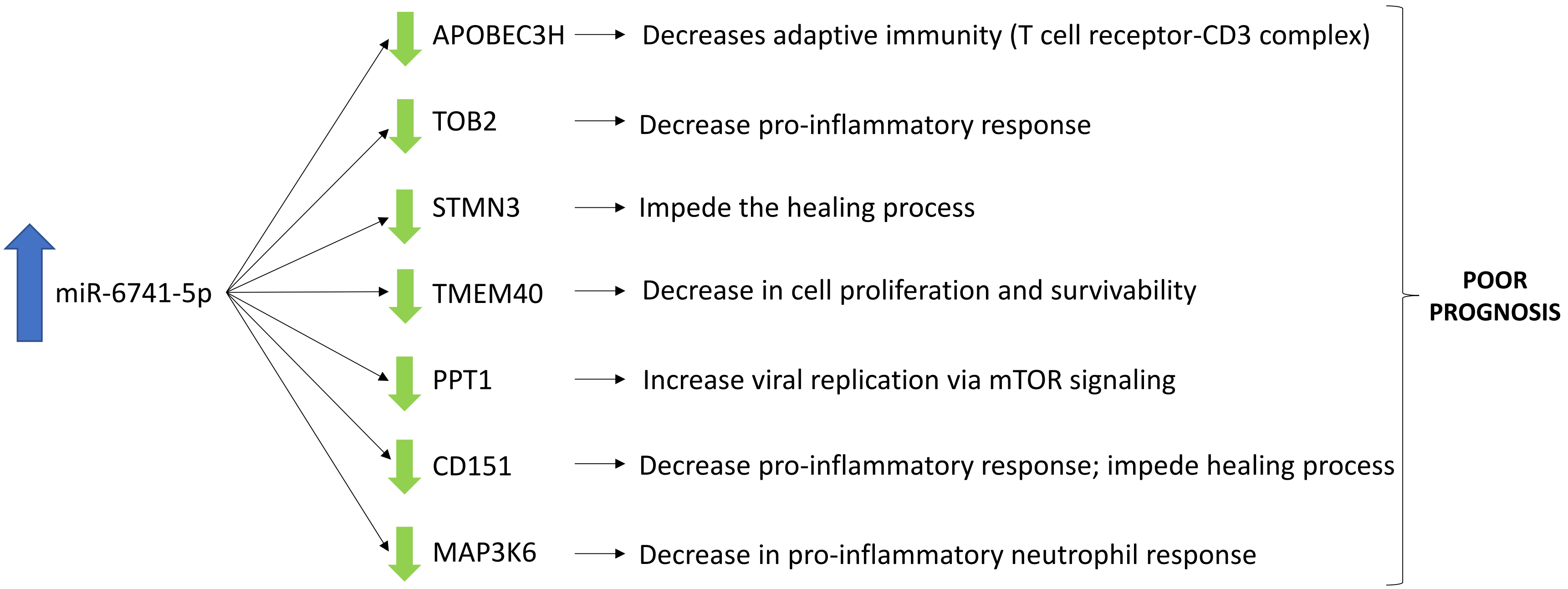

Supplemental Figure S5. The net effect of a sudden surge of miR-6741-5p in the COVID-19 patients (Jiang et al., 2020; Leonard et al., 2016; Liu et al., 2020; Mazzocca et al., 2002; Meissner et al., 2022; Mondal et al., 2022; Nair et al., 2014; Tartey et al., 2018; Yu et al., 2021).

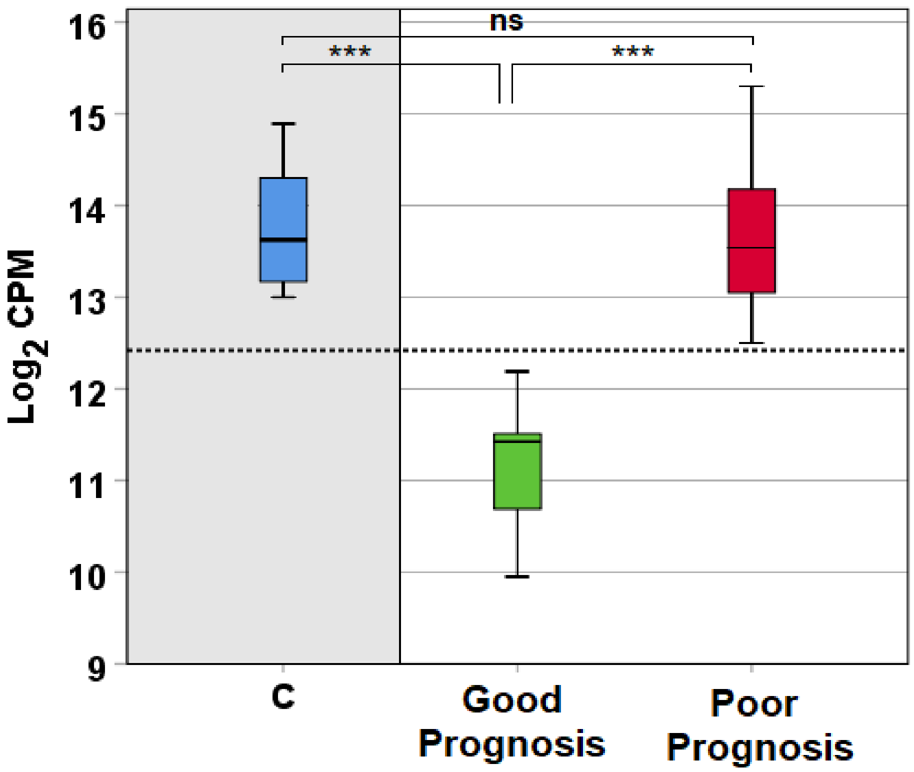

Supplemental Figure S6. Statistical comparison of the miR-6741-5p expression in healthy volunteer group (C) compared to COVID-19 patients at 48h post treatment.

Supplemental Table S2: Clinical biomarker levels during the course of treating COVID-19.

		CRP VALUES			ALC	Ferritin			D dimer			MEDICAL INTERVENTIONS					miR-6741-5p expression			
ID	Co-morbidity	D 01	D 02	D 04	D 01	D 01	D 02	D 04	D 01	D 02	D 04	Ds on MV*	D when put on MV*	Max O_2 (%)	D when given O_2?	Ds on O_2	Log$_2$ CPM value		Pred (based on CPM value)	LOS
																	D0	D02		
A	HTN, paraplegia	70	43	180	0.7	2419	6858	8098	3172	1866		0	NA	28	1	3	9.5	11.5	G	Discharged on day 4
B	HTN, DM, obesity		130		0.83				1278	75951		0	NA	100	1	3	10.4	11.5	G	Discharged on day 4
C	HTN, asthma, DM, obesity	220	202	126	0.4	205			1273			0	NA	28	1	4	8.9	10.7	G	Discharged on day 5
D	DM		43		0.92	327			3328			0	NA	100	2	15	11.4	13.2	P	Dead on day 16
E	Renal transplant, HTN, DM				0.27				876			0	NA	44	1	7	11.0	13.4	P	Discharged alive; died 3 days later
F	HTN, DM, prostate cancer	194	56		0.86				502			0	NA	100	1	22	12.1	13.8	P	Discharged on day 23
G	ESRD, HTN, DM		127	92	0.5	37372	8730		1661	9434		0	NA	28	1	11	8.2	9.9	G	Discharged on day 12
H	HTN, COPD	123			0.91				1087			0	NA	100	1	9	11.2	13.4	P	Dead on day 9
I	DM, obesity	23			1.8	39			14257			0	NA	28	1	3	8.5	10.2	G	Discharged on day 5
J	HTN, COPD, paraplegia		255		1.68	345			850			0	NA	100	1	15	9.7	12.2	P	Dead on day 15
K	HTN, CHF, cirrhosis	154	84		1.42	434			2368	12781		3	Day 6	100	1	8	8.6	12.6	P	Dead on day 8
L	HTN, DM, obesity	302	125	5	0.5	1054	1545	373	1189	6160		26	Day 6	100	1	26	11.2	12.9	P	Dead on day 26
M	HTN		52	23	0.98	1438			15696	1718		0	NA	75	1	5	11.0	11.3	G	Discharged on day 6
N	DM, obesity,	149	302	158	0.67	639	808	552	814	33960	2548	41	Day 4	75	1	56	13.2	13.8	P	Discharged on day 57

	sickle cell trait																			
O	HTN, DM, obesity	22	65	119	0.99	1835	4131	5602	1700	7338	5264	13	Day 4	100	1	16	13.0	15.3	P	Died on day 16
P	No comorbid conditions		138		0.42	751			1044	916	529	0	NA	70	1	14	12.0	12.8	P	Discharged on day 15
Q	HTN, DM, obesity	231	80		0.67	2175	1297		1079	6016		0	NA	100	1	20	13.1	14.8	P	Discharged on day 21
R	CHF, CAD	129			0.86	4160			1331			0	NA	100	1	4	9.0	10.1	G	Discharged on day 5

Legend:

D – Day(s); Day 01 is the day of admission.

Pred – Predictions

G – Good prognosis

P – Poor prognosis

MV - Mechanical ventilation

CRP range (healthy people): <5mg/l

ALC range (healthy people): 1 – 4.8k/µl

D-dimer range (healthy people): <500ng/ml

Ferritin range (healthy people): 10 – 291ng/ml

Supplemental Table S3: GO Annotations for APOBEC3H

Aspect	Function*
Cellular Component	Cytoplasm
Cellular Component	Nucleus
Molecular function	Cytidine deaminase activity
Molecular function	Deoxycytidine deaminase activity
Molecular function	RNA binding
Molecular function	Zinc ion binding
Biological process	Cytidine to uridine editing
Biological process	Defense response to virus
Biological process	DNA cytosine deamination
Biological process	DNA demethylation
Biological process	Innate immune response
Biological process	Negative regulation of single stranded viral RNA replication via double stranded DNA intermediate
Biological process	Negative regulation of transposition
Biological process	Negative regulation of viral process

Negative regulation of viral genome by APOBEC3H

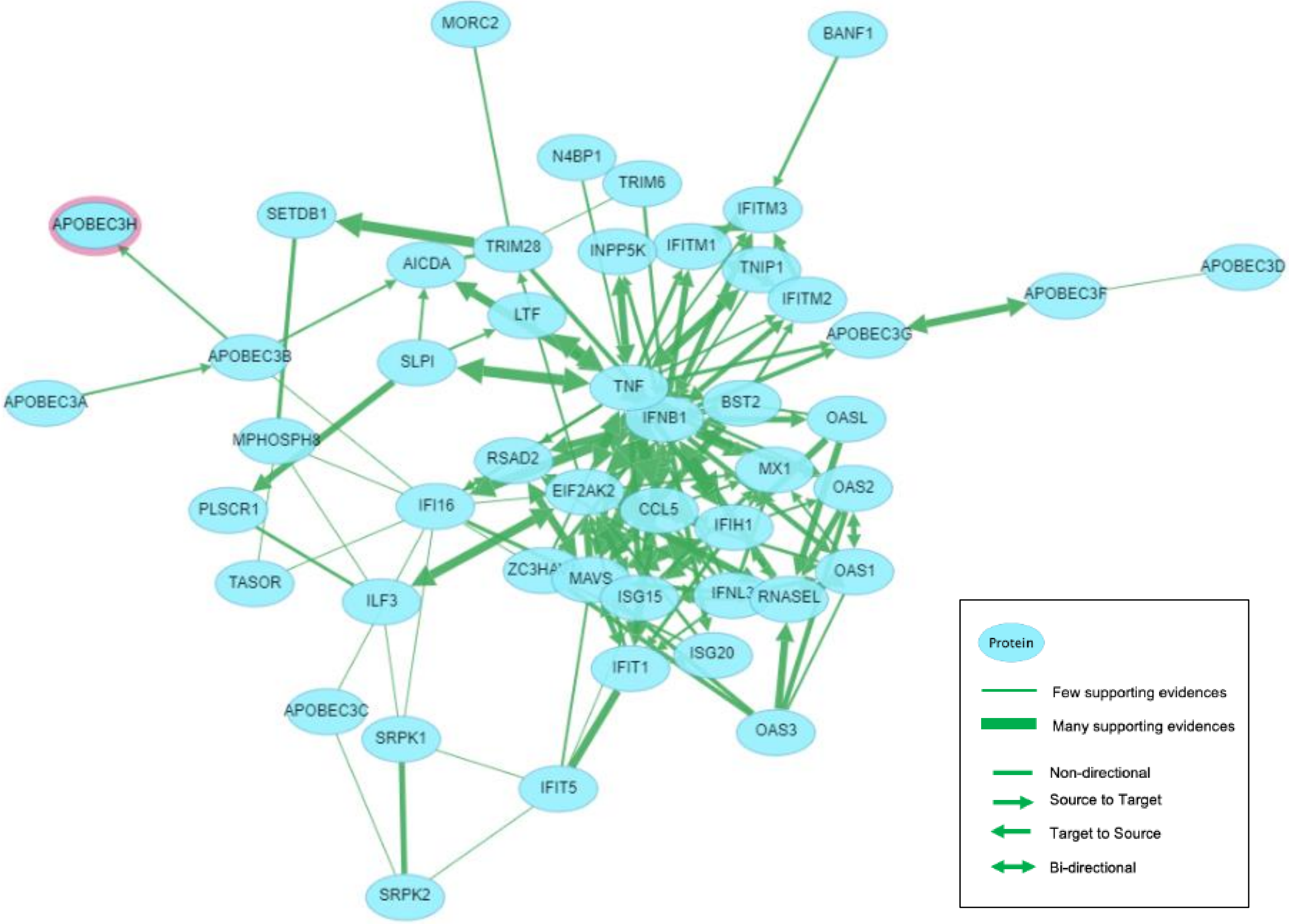

Supplemental Figure S7. Pathway map showing the negative regulation of viral genome by APOBEC3H. This network was assembled automatically by INDRA (http://indra.bio) by processing all available biomedical literature with multiple machine reading systems and integrating curated pathway databases. The network represents mechanistic interactions between genes/proteins that are associated with this GO process.
